# Newly detected mutations in the *Meq* oncogene and molecular pathotyping of very virulent Marek’s disease herpesvirus in Tunisia

**DOI:** 10.1007/s00705-020-04790-5

**Published:** 2020-09-02

**Authors:** Jihene Lachheb, Houssem Mastour, Jihene Nsiri, Khaled Kaboudi, Imed Choura, Faten Ammouna, Abdelkader Amara, Abdeljelil Ghram

**Affiliations:** 1 Laboratory of Epidemiology and Veterinary Microbiology, LR 11 IPT 03, Institut Pasteur de Tunis, Université de Tunis El Manar, Tunis, Tunisia; 2 Department of Poultry Farming and Pathology, National School of Veterinary Medicine, University of Carthage, Sidi Thabet, Tunis, Tunisia; 3 Society of Animal Nutrition (SNA), Tunis, Tunisia

## Abstract

Marek's disease (MD) is a contagious avian viral disease that is responsible for large economic losses to farmers. The disease is caused by Marek's disease virus (species *Gallid alphaherpesvirus 2*), which causes neurological lesions, immune suppression, and tumor proliferation of lymphoid cells that invade a large number of organs and tissues. Despite widespread vaccination, Marek's disease virus (MDV), has shown a continuous increase in its virulence and has acquired the ability to overcome immune responses induced by vaccines. In the present study, the oncogenic serotype MDV-1 was detected by real-time PCR in DNA samples extracted from organs developing tumor infiltrations. Identification of the pathotype based on a 132-bp tandem repeat and sequencing and phylogenetic analysis of the *Meq* gene and its encoded protein allowed classification of the isolated viruses as "very virulent", with two new and unique mutations in the *Meq* gene resulting in amino acid substitutions. Sequencing of *pp38*, *vIl-8*, *UL1* and *UL44* genes did not reveal any new mutations that were characteristic of the Tunisian isolates or correlated with virulence. These results raised concerns about the ability of HVT and CVI988 vaccines, which are currently used in Tunisia and other countries, to protect chickens against highly virulent virus strains.

## Introduction

Marek's disease (MD) is a highly contagious immunosuppressive disease that is characterized by paralysis and lymphoma development of T-cells in viscera and muscles [1]. The etiological agent is Marek's disease virus (MDV), or gallid herpesvirus 2 (GaHV-2), a member of the genus *Alphaherpesvirus*, family *Herpesviridae*, that has an enormous impact on the poultry industry [2]. The molecular basis for increased MDV virulence is currently unknown, but mutations in some regions of the viral genome, including the *Meq* (Marek's disease virus EcoRI fragment Q)*,* phosphoprotein 38 (*pp38*)*,* viral interleukin-8 (*vIL-8*)*, UL1*, and *UL44* genes have been implicated as virulence factors [3–7].

The MDV genome consists of a double-stranded linear DNA molecule of about 180 kbp. It consists of several regions, namely, long unique (UL) and unique short (US) regions flanked by long terminal repeats (TRLs) and short terminal repeats (TRSs), long internal repeats (IRLs) and short internal repeats (IRSs). The genome structure and the gene content of each region are similar in all GaHV-2 serotypes, but there are key differences [8]. Oncogenic serotype 1 is characterized by the presence of the *Meq* oncogene and other unique genes, including *pp38, vIL-8*, and *vTR*, in the repeat regions, particularly the TRL region. The *Meq* gene is considered the major factor responsible for tumorigenesis in chickens, since its deletion leads to a breakdown of T-cell transformation [9]. In addition to the *Meq* gene, which is directly responsible for transformation, the major lytic antigen pp38 has been reported to be associated with enhanced virulence and is highly expressed during lytic infection and lymphoma formation [10, 11]. There are other genes, such as *vIL-8*, whose deletion or mutation may decrease tumor development and lead to attenuation of virulence [5, 12]. Another gene that is suspected to contribute to the virulence of some hypervirulent MDV strains is the *UL1* gene, which codes for glycoprotein L (gL) [6]. This protein forms a hetero-oligomeric complex with glycoprotein H (gH-L) that plays an important role in entry of the virus into host cells and cell-to-cell infection [13] An MDV-specific cytotoxic immune response is induced by the MDV gC protein and, to a lesser extent, the gL protein [14]. The gC protein, which is encoded by the *UL44* gene, appears to be involved in virus-host interactions and is important for attenuation of virulence [7].

Due to mutations in the viral genome, the severity of disease varies among GaHV-2 isolates, leading to ever-changing pathotypes that overwhelm vaccine protection, and efforts have been made to identify new emerging pathotypes in order to ensure vaccine protection [15]. Sporadic epidemics of MD have been reported worldwide, even in vaccinated flocks [16], indicating that the virulence of MDV has increased in recent decades, and some of the more recent isolates are more pathogenic to chickens than older isolates [17, 18]. Thus, the increased use of vaccines has led the virus to evolve toward higher virulence, which can overcome the protection conferred by currently available vaccines [19]. It has been suggested that the widespread use of the CVI988 vaccine strain may have led to the emergence of new, more virulent pathotypes [20], and there are serious concerns that such pathotypes are circulating in Tunisia, especially due to the excessive use of vaccines. This poses a threat to the poultry industry and gives a higher priority to research, especially in the absence of relevant studies, particularly in Tunisia. Therefore, the main objective of this study was to determine the pathotypes of circulating GaHV-2 strains on Tunisian farms based on analysis of the *Meq*, *pp38*, *UL1*, *UL44*, and *v-IL8* genes of two MDV field isolates. This will provide a basis for the development of more effective vaccines.

## Materials and methods

### Sample collection

Two field isolates were isolated in 2016 from samples of diseased broiler chickens reared on two different farms located in the same region. The birds had developed MD symptoms, including paralysis of the legs and lethargy. At necropsy, macroscopic examination of lesions revealed tumor infiltrations in the form of nodules 2 cm in diameter, associated with several smaller nodules, in the spleen, heart and liver as well as an enlarged spleen and an enlargement and a loss of the pearly white appearance of the sciatic nerves. The liver, kidneys and heart were collected and preserved at − 80 °C until used. These birds had been vaccinated with monovalent CVI-988 vaccine when they were one day old. Specific information about these birds is shown in Table 1.

**Table 1:** Summary of the data samples

Sample	Bird number	Type	Age (weeks)	Onset of symptoms (week)	Mortality (%)	Origin
TN 1013/16	90	Broiler	18	7th	11.1	Cherfech-Ariana
TN 1014/16	40	Broiler	6	4th	30	Borjyoussef-Ariana

### Virus purification, isolation, and identification

Organs were homogenized in a blender in the presence of Dulbecco's modified Eagle medium (DMEM) containing 5% antibiotics. The mixture was clarified by centrifugation at 1500 rpm for 15 min and passed through a 0.22-µm filter. A volume of 3 ml of each viral suspension was placed on the top of a layer of sucrose and ultracentrifuged at 40,000 rpm for 2 h. The resulting virus particle layer was recovered, suspended in DMEM, and stored at -80 °C. Primary cultures of chicken embryo fibroblasts (CEFs) were prepared according to standard protocols [21].

A volume of 100 μl of purified virus suspension was used to inoculate the cells, which were then incubated at 37 °C for 10 days. The cells were harvested after the appearance of a cytopathic effect (CPE) and lysed by two cycles of freezing and thawing.

### DNA extraction and detection of the viral genome by PCR

Extraction of viral DNA from the internal organs was performed using an EasyPure Genomic DNA Kit (TransGen Biotech) according to the manufacturer's instructions. The highly conserved MDV-1-A antigen region of the glycoprotein C gene was targeted using specific primers described by Zhu et al. [22]. Real-time amplification reactions were carried out using a Swift Spectrum 48 Thermal Cycler (ESCO) and using KAPA SYBR FAST qPCR Master Mix using the following reaction conditions: incubation for 3 min at 95 °C to activate the enzyme, followed by 40 cycles of 3 s at 95 °C and 1 min at 60 °C. Extracted DNA from the CVI 988 Rispens vaccine and a negative control were included in each experiment. The extracted DNA was also tested for avian leukosis virus subgroups J and K, using real-time PCR to confirm specificity [23, 24].

### Virus pathotyping by conventional PCR

DNA extracted from the tissues was amplified using primer pairs described by Tian et al*.* [4], Becker et al*.* [25], and Hassanin et al. [26]*.* for the *Meq, vIL-8*, *pp38, UL1*, and *UL44* genes and the 132-bp tandem repeat regions, generating fragments of 1094, 830, 1006, 576, 1506 and 434 bp, respectively. The reaction mixtures were optimized using a KAPA Taq PCR kit with 2 μl (0.2 μM) of each of the two primers for each gene, 5 μl of 10 × KAPA Taq buffer, 1 μl of dNTP mix (0.2 mM), 0.2 μl of Taq polymerase (0.02 U/μl), 2 μl of DNA extract, and nuclease-free water in a final volume of 50 μl. Amplification was carried out in a Bio-Rad T100 thermal cycler, using the following reaction steps: denaturation for 4 min at 95 °C, followed by 35 cycles consisting of a second denaturation for 1 min at 94 °C, hybridization of the primer pairs for *Meq, vIL-8*, *pp38*, 132-bp tandem repeat, *UL1,* and *UL44* for 1 min at 59, 56, 60, 52, 55 and 55 °C, respectively, and elongation for 1 min/kb at 72 °C, followed by a final elongation for 10 min at 72 °C. The amplified products were separated by electrophoresis on a 2% agarose gel containing 0.5 mg of ethidium bromide (BET, Sigma) per ml, visualized using a Gel Doc 2000 system (Bio-Rad), and used for sequencing.

### Sequencing and phylogenetic analysis

To analyse the *Meq, v-IL8*, *pp38,* and *UL1* and *UL44* genes and the 132*-*bp tandem repeat of MDV, PCR products were purified using a Gel and PCR Extraction Kit (BioBASIC) and sequenced using an ABI PRISM 3500 Genetic Analyzer (Applied Biosystems). A multiple alignment of the sequences of the Tunisian isolates with reference sequences downloaded from the GenBank database was performed using the programs Bioedit (version 7.2.5.0) and ClustalW. Phylogenetic analysis was done by the maximum-likelihood method in MEGA6 (Version 6) and evaluated statistically by analyzing 100 bootstrap replications. The genome sequences of the GaHV-2 isolates were submitted to the GenBank database and assigned the accession numbers KY113150 and MK041219 for *Meq*, KY113151 and MK058702 for *vIL8,* KY113152 and MK058701 for the 132-bp tandem repeat, MN128713 and MN128714 for *pp38*, MN018231 and MN030639 for *UL1*, and MN480312 and MN480313 for *UL44* of TN1013/16 and TN1014/16, respectively.

## Results

### Virological analysis

#### Isolation and identification of viruses

After two passages of organ suspensions in chicken embryo fibroblasts, a cytopathic effect was observed, with refractive rounded cells clustering together, as is typical for Marek's disease virus. During the third passage, a progressive development of CPE in the form of rounded cells was observed. In the fourth passage, clear CPE was observed as early as day 4 post-inoculation in the form of aggregated rounded cells with characteristic cytoplasmic extensions. No detached cells were observed during the four passages. Virus titration of both isolates, performed in a Vero cell culture in a 96-well microtiter plate with flat-bottomed wells, resulted in TCID_50_ titers of 10^6.30^/ml, and 10^5.4^/ml for TN1013/16 and TN1014/16, respectively.

#### Macroscopic lesions in embryos

All chicken embryos that were infected with 0.2 ml of GaHV-2 at a concentration of 10^5^ TCID_50_/ml died on day 9, and necropsy revealed macroscopic lesions showing atrophy, liver pallor, and leg malformations. In addition, small white vesicles were observed on the allantoic membrane.

### Molecular analysis

#### Detection of the viral genome

The results of SYBR Green real-time PCR amplification, carried out on organ suspensions, revealed a single type of amplicon for both samples with a specific melting temperature of 82.4 °C, confirming the presence of the GaHV-2 genome.

#### Sequence alignment and phylogeny of the *Meq* gene

Complete nucleotide sequences of *Meq* oncogene as well as their encoded protein sequences were analyzed. The *Meq* gene sequences of the isolates TN1013/16 and TN1014/16 were 100% identical. The sequences of *Meq* gene of various strains having different degrees of attenuation (att), including the currently used vaccine strain (CVI988), and moderately virulent (mv), virulent (v), very virulent (vv) and very virulent plus (vv +) strains from different countries, were selected and included in this analysis. Alignment of nucleotide sequences of all these strains showed that the *Meq* oncogene sequences of the Tunisian isolates shared the highest percentage of sequence identity with vv and vv + strains from Italy (100%) and China (99% and 99.6%, respectively). The lowest sequence similarity was observed with strains from the USA and Australia, with 98.8% to 99.4% and 84.4% to 84.6% identity, respectively.

Moreover, the *Meq* oncogene sequences of both TN1013/16 and TN1014/16 shared 98.8–99%, 84.4–99.4%, 84.5–99.3%, and 84.3–94.6% identity with the vv + , vv, v, and attenuated vaccine strains, respectively. An alignment of the predicted amino acid sequences indicated that the proteins of both Tunisian isolates showed the lowest percentage of identity (83%) to the current vaccine strain CVI988 (DQ534538) and were 100% identical to that of the Italian strain GaHV-2/Italy/Ck/674/16 (MK139667) and 99.8% identical to those of the highly virulent Chinese strains GXY2 (EF546430), YLO (DQ174459), and GX070060 (EU427303).

An exhaustive comparison of the nucleotide sequences of *Meq* oncogenes of the Tunisian isolates with those of the reference strains in the GenBank database, showed the presence of the substitutions T > C and G > A at positions 328 and 811, respectively, which are specific to the Tunisian and the Italian isolates.

The protein encoded by the *Meq* gene was found to be 339 amino acids long in most virulent strains, except for the Australian strains, 398 amino acids long in the attenuated and v and vv Australian strains, and 399 amino acids long in the vaccine strain CVI988. The TN1013/16 and TN1014/16 isolates both contained arginine instead of cysteine at position 110 and glycine at position 271.

**Fig. 1:**
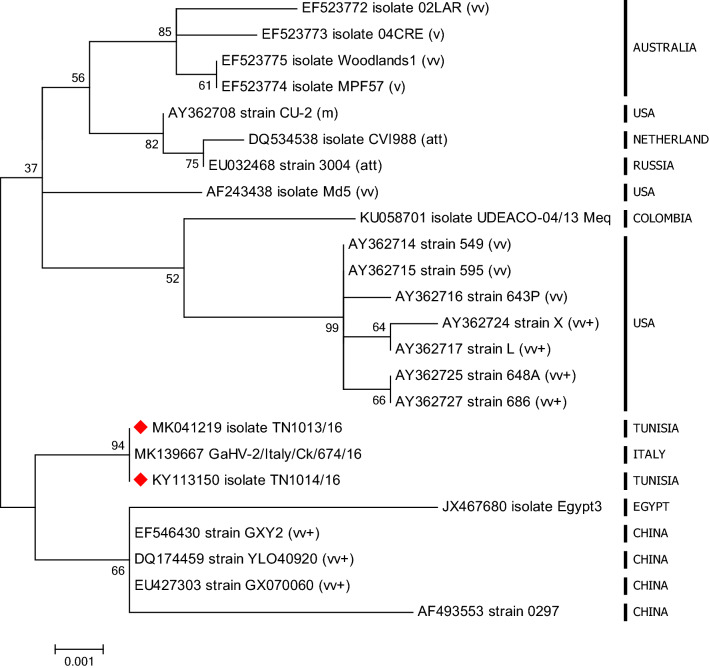
Phylogenetic tree of GaHV-2 based on the *Meq* gene. The tree was constructed using maximum-likelihood algorithm implemented in MEGA 6.0.6 with 1000 bootstrap replicates. Tunisian isolates are indicated by red diamonds

A phylogenetic analysis based on the nucleotide sequences of the *Meq* gene, using the maximum-likelihood algorithm, is presented in Fig. 1. The phylogenetic tree showed that the GaHV-2 isolates are divided into two heterogeneous clusters. Cluster 1 is composed of two branches, the first of which is composed of strains with different levels of virulence and from different geographic locations and includes attenuated strains such as strain 3004 from Russia and strain CVI988 from the Netherlands, the mv-like CU-2 from the USA, and the v-like 04CRE and vv-like 02LAR from Australia. The second branch is composed of vv and vv + American and Colombian strains.

The second cluster is composed of two branches, the first of which includes the Tunisian and Italian strains, and the second of which includes Egyptian and vv + Chinese strains.

#### Pathotyping based on the sequence of Meq protein

Molecular determination of the pathotype of GaHV-2 strains is typically based on the sequence of the Meq protein, in particular, the number of proline repeat (PPPP) motifs. The v, vv and vv + strains are characterized by the presence of two to five repeats [27]. The v strains have five repeats (04CRE [EF523773], MPF57 [EF523774]); the vv strains may also have five (RB1B [AY243332], 02LAR [EF523772], Woodlands1 [EF523775]) or four (Md5 [AF243438] and GaHV-2/Italy/Ck/674/16 [MK139667]), and the vv + strains may have three (GXY2 [EF546430], YLO40920 [DQ174459], GX070060 [EU427303], 0297 [AF493553]) or even two PPPP motifs (686 [AY362715], 686A [AY362725], GaHV-2/Italy/Ck/855/17 [MK139678]).

The TN1013/16 and TN1014/16 isolates have four PPPP motifs, like the majority of the vv strains, such as Md5. This suggests that the Tunisian isolates are probably vv strains.

A low proline percentage has been shown to correlate with high virulence [27]. We therefore calculated the proline percentage of the Tunisian isolates and compared them to the reference strains of different pathotypes (Table 2). The Meq protein of isolates TN1013/16 and TN1014/16 was composed of only 21.18% proline, which represents a relatively small percentage compared to that of mv and v strains, and even with the majority of vv strains. This reinforces the hypothesis that these isolates are hypervirulent vv or vv + .

**Table 2:** Number of PPPP signatures and proline percentage in different GaHV-2 pathotypes

Isolate	Country	Accession number	Size of Meq (aa)	Proline %	Number of PPPP repeats	Pathotype
CVI988	Netherlands	DQ534538	399	23.3	8	AttMDV
JM/102 W	United States	DQ534539	399	23.1	6	vMDV
MPF57	Australia	EF523771	398	22.9	5	vMDV
RB1B	United States	AY243332	339	21.5	5	vvMDV
Md5	United States	AF243438	339	21.3	4	vvMDV
TN1013/16	Tunisia	MK041219	339	21.18	4	vvMDV
TN1014/16	Tunisia	KY113150	339	21.18	4	vvMDV
648A	United States	AY362725	339	20.9	2	vv + MDV
GaHV-2/Italy/Ck/855/17	Italy	MK139678	298	19.4	2	–
GaHV-2/Italy/Ck/674/16	Italy	MK139667	339	21.18	4	–

The length of the Meq proteins of the TN1013/16 and TN1014/16 isolates was compared to those of reference strains of known pathotypes (Table 2). All of the hypervirulent strains that were analyzed were found to have a Meq protein of 339 aa. This was the case for the TN1013/16 and TN1014/16 isolates, again suggesting that these isolates are pathogenic.

#### Pathotyping based on the 132-bp tandem repeat region

**Fig. 2:**
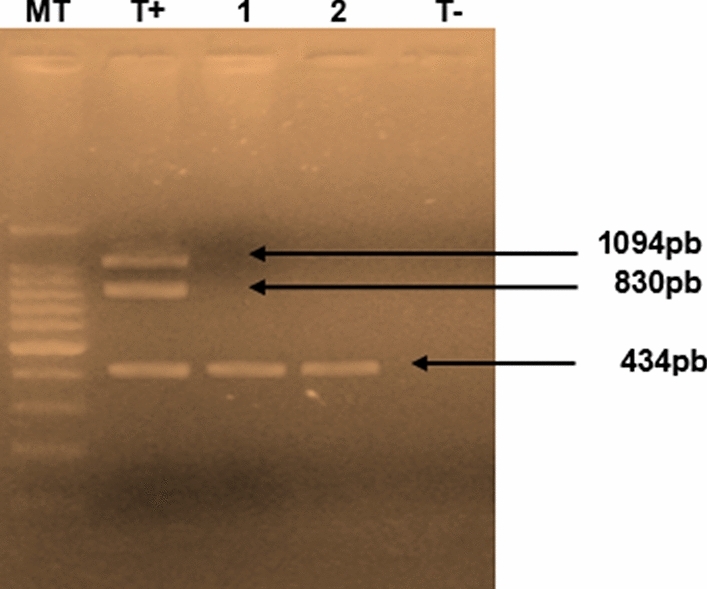
Amplification profile of the 132-bp tandem repeat on a 1.5% agarose gel. MT, 100-bp size marker; T + , vaccine (CVI988 Rispens); 1, sample TN1013/16; 2, sample TN1014/16; T-, negative control

The 132-bp tandem repeat region, present in the BamHI-H segment of the viral genome, may be present one or more copies in MDV strains. The number of repetitions of one sequence is an indicator of the pathogenicity of the virus [25]. The electrophoretic profiles of strains from this study showed amplification of a 434-bp fragment from both TN1013/16 and TN1014/16, which can be explained by the presence of two copies of the 132-bp tandem repeat sequence, thus confirming their pathogenicity and suggesting their high virulence. On the other hand, the attenuated vaccine strain (CVI988 Rispens) showed an electrophoretic profile with three bands of 434, 830 and 1094 bp, indicating the presence of two, five and seven copies, respectively, of the 132-bp tandem repeat in the genome of the vaccine strain (Fig. 2).

#### Sequence comparisons and phylogeny of the *VIL-8, pp38, UL1 *and *UL44* genes

Sequencing of the amplified products of each field isolate yielded sequences of 682, 1006, 585 and 1506 bp, corresponding to the complete size of the *vIl-8, pp38, UL1* and *UL44* genes*,* respectively. These gene sequences were 100% identical in TN1013/16 and TN1014/16.

Multiple alignments of amino acid sequences showed variability at positions 4 and 31 of the vIL-8 protein. The v and vv strains BY (HQ638179), LS (HQ638183), SD2012 (KC511812), MS01 (HQ638184) and YA (HQ638196) have a serine residue at position 4, while the Tunisian and other strains, including the v strain GA, vv and vv + strains 584a (DQ534532) and 648a (AF489277), have a leucine residue at this position. The Tunisian isolates and the vv Chinese and American strains J1 (HQ638182), Md5 (AF489275), 595 (DQ534533), RB1B (EF523390) and vv + strains 584a (DQ534532) and 648a (JQ809692) have aspartic acid residue at position 31. However, the v strains LMS (JQ314003), LS (HQ638183), Ind/TN12/02 (KP644422) and Ind/TN11/01(KP644421) and the vv strain Ind/KA12/02 (KP644422) have a glycine at the same position. Thus, the four amino acids at positions 4 and 31 do not appear to determine the degree of virulence of the strain.

Analysis of the *pp38* gene showed that the Tunisian isolates as well as the majority of the other strains, regardless of their degree of virulence, have a glycine at position 109. Strains with a glutamate at this position have different degrees of virulence. A nucleotide sequence alignment of the *pp38* genes of the Tunisian isolates and the reference strains showed 100% identity to the Chinese v strain SD2012 (KC511813) and mv strain CU-2 (EU499381) from the USA.

Glycoprotein L exhibited a high degree of sequence stability, and its deduced amino acid sequence was 97.9% to 100% identical to those of reference strains from other countries. Our strains did not show the deletion of 12 nucleotides in the cleavage site at positions 54–65, that were reported previously for Japanese (Gifu 2 (LC208808) and American TK (AY129998) and 06-L (AY130000) strains.

A phylogenetic analysis based on the *UL1* gene also showed that the isolates were divided into two distinct phylogenetic groups. The first group included the Tunisian isolates TN1013/16 and TN1014/16 as well as all other strains with different degree of virulence from different countries, while the second branch included vv and vv + strains from the United States (TK and L) and Japan (Gifu2) with a deletion of 12 nucleotides.

The *UL44* gene was found to be 100% identical to those of the Chinese vv strain LMS and the American strain Md5. The *UL44* gene displayed high sequence conservation, regardless of virulence and was the most conserved among sequenced genes in this study.

A phylogenetic tree based on the nucleotide sequences of the *vIL-8, pp38, UL1* and *UL44* genes showed that TN1013/16 and TN1014/16 both belong to a heterogenous group, with different levels of virulence.

## Discussion

In recent years, numerous isolations of vv and vv + MDV strains from vaccinated chickens have been reported, and very common forms of proliferative lymphatic diseases have been described [20]. The virulence of MDV isolates has increased in recent decades, and some recently isolated vv and vv + strains have been shown to be more pathogenic for chickens than the older ones [28]. Recombination events among MDV strains could be among the mechanisms by which virulence increases [29]. It is therefore justified to be concerned about new emerging strains that can break vaccine protection.

Some GaHV-2-specific genes, including *Meq, pp38, UL1, UL44* and *vIL-8*, contain sequence differences that are associated with oncogenicity, viral pathogenicity and virulence [26, 30]. In this study, to better understand the evolution of Tunisian isolates, we studied the pathotypes of MDV strains circulating on farms based on the *Meq, vIL-8, pp38, UL1* and *UL44* genes.

The *Meq* gene has been studied extensively and appears to correlate with virulence [3]. Indeed, it has been reported that a change of serine to alanine at position 71 is a characteristic of hypervirulent strains of MDV [3]. This has been observed in the Chinese vv isolate SD2012-1 (KC511815) and vv + isolate JZ2014 (KP144355), the American isolates N (AY362718), W (AY362723), 584A (DQ534532), L (AY362717) and X (AY362724), and the Tunisian isolates TN1013/16 (MK041219) and TN1014/16 (KY113150). However, some vv strains, such as 02 LAR (EF523772) and Woodlands1 (EF523775), have a serine at this position, casting doubt on whether the presence of this mutation is a reliable criterion for classifying MDV pathotypes.

The substitution of glutamate by leucine at position 77 has been reported by Shamblin et al. [3]*.* to be a feature of hypervirulent strains. However, isolate JZ2014 (KP144355), the Colombian isolates UDEACO04 (KU058701) and SD2012-1 (KC511815), and the Tunisian isolatesTN1013/16 (MK041219) and TN1014/16 (KY113150), have glutamic acid at position 77 but are classified as vv and vv + strains. This suggests that the presence of glutamic acid at position 77 is not necessarily a characteristic of low-virulence MDV strains. On the other hand, the presence of the amino acids R_119_, Q_153_ and A_176_, has been shown to be a common feature of the American vv isolates 549 (AY362714) and 595 (AY362715) and the American vv + isolates N, L, X, 584A, and 684A (AY362725), in contrast to a report by Tian et al*.* [4], who suggested that these mutations are unique in vv + strains. However, the Tunisian isolates share R_119_, and P_176_ as well as P_153_.

MDV strains with high virulence have been shown to contain mutations at the second position of the proline-rich region (PRR): PPPP > P (Q / A / R) PP [3]. Attenuated strains have more PPPP motifs, whereas most virulent strains have more interrupted motifs. Analysis of the Meq protein amino acid sequences of theTN1013/16 and TN1014/16 isolates revealed a point mutation in the proline-rich region at position 217, interrupting the PPPP motif. A variable number of PPPP motifs in the central PRR of the Meq protein was observed in low-virulence strains, and in fact, eight repeats were detected in the vaccine strain CVI988, and nine and 10 in the Italian strains GaHV-2/Italy/Ck/847/15 and GaHV-2/Italy/Ck/847/17, respectively [31]. In contrast, hypervirulent strains have low number of these motifs, and the smallest number was observed in the vv + strain 648A, with only two motifs. The hypervirulent vv and vv + strains contained interruptions in the proline repeats at the second position (P > Q_153_, P > A_176_ and P > A_276_), and the largest number of such interruptions was observed in the most virulent strains [32]. Both TN1013/16 and TN1014/16 contained only the interruption at position 217 that is present in all virulent strains except RB1B.

On the other hand, Wajid et al. [33]*.* have suggested a correlation between the proline content and the virulence of emerging strains. Analysis of reference strains showed that this parameter is inversely proportional to virulence. In this case, this percentage is around 21.18% in the Tunisian isolates, reinforcing the hypothesis that they are very virulent.

Mutations at positions 110 and 271 appear to be characteristic of Tunisian and Italian isolates [31]. Such mutations and their overall sequence similarity might suggest that migratory birds such as ducks and white footed geese play a role in transporting the virus from one country to another [34]. The genetic divergence of MDV strains in the *Meq* gene is more obvious than in the *vIL-8, pp38, UL1* and *UL44* genes, making the *Meq* gene most appropriate for phylogenetic analysis.

In addition, the *vIL-8* gene, located in the long repeat (RL) region, was initially identified as a spliced ​​variant of the *Meq* gene [35]. It is highly conserved in all strains but with some variability at sites 4 and 31 of the vIL-8 protein. However, these mutations are not characteristic of virulent strains.

As reported by Shamblin et al*.* [3], we did find some mutations in the *UL1* and *UL44* genes that strictly correlate with the virulence level. These mutations map to the putative signal cleavage site of the *UL1* genes, and are found in four out of 11 vv + MDVs, but also in one vvMDV (643P), indicating that it does not correlate with enhanced virulence.

Although several early genes have been shown to be essential for viral replication, and thus for initiating the transformation pathway and subsequent development of clinical signs of MD, the *Meq* gene is considered to be the primary oncogene of MDV, with other genes serving as auxiliary factors [10]. Similarly, although the *Meq* gene is mainly expressed during the latent state, studies have indicated that the *Meq* gene can also be expressed early in infection [10], suggesting that variations in the *Meq* gene sequence can have a significant influence on virulence. However, previous gene sequencing studies have not revealed mutations in other genes that are consistently correlated with virulence [3].

Our findings showed that there is significant polymorphism in the MDV *Meq* gene, which is a key gene in the induction of lymphoid tumors. The Tunisian isolates had two point mutations in the *Meq* gene that might be associated with virulence. In addition, the overall proline content and pattern of PPPP repeats showed strong correlation with the virulent pathotype. Although the virulence of a specific isolate is unlikely to be determined solely by mutations in a single gene, the results of our work suggest that *Meq* gene sequencing and protein analysis can provide a useful indication of the virulence of these isolates. However, confirmation of the pathotype requires in vivo experiments.

The CVI988 vaccine is widely used around the world. In China, it has been noted that immune failure tends to occur in only a few chickens. A number of vv strains have been isolated from different parts of China [36], raising the question whether the currently available vaccines are able to protect chickens against these highly virulent strains.

The weak protective effect of MDV vaccines has led to MD outbreaks [16], and the emergence of MDV strains with increasing virulence is therefore an important issue for the poultry industry.

## Conclusion

Our results have made it possible, for the first time, to be aware of the presence of highly virulent strains circulating on Tunisian farms. Variations in the Meq protein sequence, the composition and the number of PPPP motifs present in the PRR region, and the size of the Meq protein can serve as indicators for determining the pathotype of MDV isolates. Simple sequencing of the *Meq* gene can be used to identify any changes in the virus pathotype and consequently to prevent a potential **??**epidemic**??** following the emergence of new more virulent pathotype.

In addition, we found two mutations in the Tunisian isolates that had not been observed in any other strain with sequences in the GenBank database until 2019, when Mescolini et al*.* [31] found the same mutations. In addition, the presence of two copies of the 132-bp tandem repeat segment has made it possible to characterize these strains and confirm their pathogenicity. This will be useful for developing rapid diagnostic methods for detection and assessment of the pathogenicity of circulating MDV strains.

## Data Availability

Data and materials from the current study are available from the corresponding author on reasonable request.
